# Clinical Correlates of Alzheimer's Disease Cerebrospinal Fluid Analytes in Primary Progressive Aphasia

**DOI:** 10.3389/fneur.2019.00485

**Published:** 2019-05-10

**Authors:** Catherine Norise, Molly Ungrady, Amy Halpin, Charles Jester, Corey T. McMillan, David J. Irwin, Katheryn A. Cousins, Murray Grossman

**Affiliations:** Department of Neurology and Penn FTD Center, University of Pennsylvania, Philadelphia, PA, United States

**Keywords:** primary progressive aphasia (ppa), PPA, lvPPA, logopenic variant primary progressive aphasia, CSF, logopenic primary progressive aphasia, cerebrospinal fluid

## Abstract

**Background:** While primary progressive aphasia (PPA) is associated with frontotemporal lobar degeneration (FTLD) pathology due to tau or TDP, clinical-pathological studies also demonstrate many cases have Alzheimer's disease (AD) pathology. The logopenic variant of PPA (lvPPA) is most often associated with AD pathology, but this has proven to be the least reliable PPA to diagnose using published clinical criteria. In this study, we used cerebrospinal fluid (CSF) analytes to identify patients with likely AD pathology, and relate phenotypic features of lvPPA to CSF.

**Methods:** We studied 46 PPA patients who had available CSF analytes, including 26 with a clinical diagnosis of lvPPA, 9 with non-fluent/agrammatic variant (naPPA), and 11 with semantic variant (svPPA). We identified patients with likely AD pathology using amyloid-beta 1–42 (Aβ_1−42_) < 192 pg/ml and assessed MRI gray matter atrophy in these patients.

**Results:** We found that 23 (49%) of 46 PPA patients have a low CSF Aβ_1−42_ level consistent with AD pathology. Twenty-one (91%) of 23 patients had a lvPPA phenotype, and 18 (79%) of 23 cases with an elevated CSF Aβ_1−42_ level did not have a lvPPA phenotype. Patients with a lvPPA phenotype demonstrated GM atrophy in the left lateral temporal lobe, and this was also seen in those with a CSF Aβ_1−42_ level < 192 pg/ml.

**Conclusion:** The lvPPA clinical phenotype may be a useful screen for CSF analytes that are a surrogate for likely AD pathology, and may help establish eligibility of these patients for disease-modifying treatment trials.

## Introduction

Primary progressive aphasia (PPA) refers to a syndrome of declining language ability that results from a neurodegenerative disease. Three variants of PPA have been identified: non-fluent/agrammatic (naPPA), semantic (svPPA), and logopenic (lvPPA) (1). It is valuable to identify these variants of PPA because there is a statistical association of a PPA variant with a specific underlying pathology (2, 3). There is broad agreement on the clinical characteristics that distinguish naPPA and svPPA (4–7). The typical clinical presentation of naPPA involves effortful speech, agrammatism, and motor speech errors known as apraxia of speech (6, 8, 9), and autopsy studies have indicated that naPPA is often associated with frontotemporal lobar degeneration (FTLD) with underlying FTLD-tau pathology (2, 3). svPPA is characterized by impairments in naming and single-word comprehension (9, 10), and svPPA is often associated with underlying FTLD-TDP pathology (3, 11). However, compared to naPPA and svPPA, lvPPA cases are more challenging to identify clinically (4–7, 12). lvPPA is said to be characterized by difficulty with repetition and lexical retrieval (1, 13), but criteria for a repetition impairment have been challenging to identify and lexical retrieval deficits are ubiquitous among patients with PPA. Correspondingly, there has been some variability in the pathology found in patients with a clinical diagnosis of lvPPA, although lvPPA is often associated with Alzheimer's disease (AD) pathology (2, 3, 11, 14).

Since cerebrospinal fluid (CSF) analytes have been shown to serve as a sensitive biomarker for AD pathology (15), this study examined the usefulness of CSF markers in identifying likely AD pathology in individuals with PPA. Specifically, we used the CSF level of beta-amyloid 1–42 (Aß_1−42_) to identify PPA patients with likely AD pathology, and assessed whether this corresponds to PPA patients with a lvPPA phenotype. Several previous studies have examined Aß_1−42_ in PPA. In one study, patients with a clinical diagnosis of lvPPA had lower Aß_1−42_ levels than controls and naPPA patients (16). In a large, multi-center cohort of PPA patients with pathology determined by CSF or positron emission tomography (PET) molecular markers or autopsy findings, 86% of lvPPA patients had findings consistent with Aß pathology (17). Here we contrasted PPA patients with low Aß_1−42_ relative to those with elevated Aß_1−42_ levels, and independently verified diagnosis in the PPA patient groups with MRI analyses consistent with published imaging data.

## Methods

### Patients

Patients included for this study were seen in the Department of Neurology out-patient clinic of the Penn FTD Center and data were retrieved from The Integrated Neurodegenerative Disease Database (INDD) (18) at the University of Pennsylvania. All patients had a clinical diagnosis of PPA involving prominent language difficulty and minimal evidence of impairment in other cognitive domains (19) based on a semi-structured clinical history, a complete neurological evaluation, and a detailed mental status assessment. From among 131 individuals with a diagnosis of PPA in INDD who also had CSF data, we restricted participants in the current study to those that met strict clinical diagnostic criteria for a specific variant of PPA (1) as adapted recently for lvPPA (14). In this study, a diagnosis of lvPPA included deficits of word-finding difficulty in continuous speech with impaired phonological loop functioning measured by a short forward digit span. Using these criteria, 46 patients were included for analysis (lvPPA: *n* = 26, svPPA: *n* = 11, naPPA: *n* = 9) (Table 1). All patients were native English-speakers with a high school education, and were matched for age, education, and disease duration at lumbar puncture. Patients were excluded if elementary neurological features such as bulbar motor weakness or extrapyramidal disease suggesting a likely pathologic diagnosis were present or other medical, psychiatric, or neurological conditions (e.g., head trauma, stroke, hydrocephalus) were present that could clinically resemble PPA. Al MRIs were clinically inspected to insure that there was minimal small vessel ischemic disease (Fazekas ≤ grade 1), and there was no evidence of cerebral microbleeds on any of the MRIs. All mutation carriers were excluded. An autopsy evaluation was available for three cases. Two cases with an lvPPA phenotype had a CSF Aß_1−42_ level that was < 192 pg/mL and AD pathology. One autopsy case with FTLD-Tau pathology had a CSF of Aß_1−42_ level that was >192 pg/mL and did not have a lvPPA phenotype. Some of these cases have participated in other CSF biomarker studies.

**Table 1:** Patient demographic characteristics and cerebrospinal fluid analyte levels.

	**lvPPA**	**naPPA**	**svPPA**
Gender (F/M)	16/10	2/7	7/4
Age at CSF (years)	62.6 (8.2)	64.2 (9.4)	63.1 (5.6)
Education (years)	15.8 (3.4)	15.3 (3.2)	16.7 (2.5)
Disease duration AT CSF (years)	2.7 (1.6)	2.7 (2.1)	2.4 (1.9)
Aβ_1−42_ pg/mL^a^	175.8 (105.0)	341.3 (176.3)^b^	323.2 (122.0)^b^
Phosphorylated tau pg/mL^a^	38.7 (24.4)	22.6 (20.1)^b^	18.1 (16.9)^b^
Total tau pg/mL	137.8 (139.1)	118.3 (143.5)	85.7 (104.8)
Total tau/Aβ_1−42_ pg/mL^a^	0.005 (0.004)	0.001 (0.001)^b^	0.001 (0.000)^b^

*lvPPA, logopenic variant primary progressive aphasia; naPPA, non-fluent/ agrammatic variant primary progressive aphasia; svPPA, semantic variant primary progressive aphasia; Low Abeta (Aß_42_ level < 192 pg/mL); High Abeta (Aß_42_ level >192 pg/mL)*.

a *Significant difference between groups, according to Kruskal-Wallis test (all p ≤ 0.02)*.

b *Differ significantly from lvPPA group (all p ≤ 0.017)*.

We assessed the neuropsychological profile for each subtype of PPA (Table 2). We evaluated the patients' performance on naming by using the Boston Naming Test (BNT) (20), function of the phonologic loop using forward digit span {Antonio:to}, executive functioning as determined by the amount of words beginning with the letter “F” in 1 min {Tombaugh:tg}, and episodic memory by the immediate and delayed recall of the Craft story {Monsell:uu}. Shapiro tests were used to check for a normal or abnormal distribution of the data. The lvPPA group had neuropsychological data that was not normally distributed, so we performed Kruskal–Wallis tests to assess the differences of each group. Analyses showed a significant difference in BNT, forward digit span, and memory. No significant differences were observed between groups for words per minute or F words per minute. Pair-wise group differences with Mann–Whitney U are summarized in Table 2.

**Table 2:** Patient neuropsychological profiles by phenotype.

	**lvPPA**			**naPPA**			**svPPA**		
**Neuropsychological tasks**	**Score (SD)**	***N***	**Time from CSF (SD) (m)**	**Score (SD)**	***N***	**Time from CSF (SD) (m)**	**Score (SD)**	***N***	**Time from CSF (SD) (m)**
Boston naming test^a^	19.95 (9.29)	19	0.73 (2.31)	23.78 (6.03)	9	0.11 (0.33)	6.63 (6.12)^b^	11	3.82 (11.70)
Digit span forward^a^	4.1 (0.88)	10	2.9 (6.15)	3.2 (1.92)	5	16 (22.31)	6.86 (1.46)^b^	7	5.57 (9.73)
F Words/minute	6.39 (4.77)	18	7.63 (12.54)	6.13 (3.14)	8	0.5 (0.76)	9 (5.42)	11	3.27 (6.90)
Craft story Immediate recall^a^	4.00 (2.45)	7	26.57 (19.15)	18.33 (0.07)^b^	3	47.67 (0.58)	5.50 (3.51)	6	39.83 (18.11)
Craft story delayed recall^a^	4.43 (2.30)	7	26.57 (19.15)	13.33 (9.29)^b^	3	47.67 (0.58)	5.33 (2.88)	6	39.83 (18.11)

*lvPPA, logopenic variant primary progressive aphasia; naPPA, non-fluent agrammatic variant primary progressive aphasia; svPPA, semantic variant primary progressive aphasia*.

a Significant Kruskal–Wallis test (p < 0.02);

b *Significant Mann–Whitney U group difference relative to lvPPA (p < = 0.017)*.

### Standard Protocol Approvals, Registrations, and Patient Consents

All procedures, including CSF collection and MRI, involved participation in an informed consent procedure, and were performed in accordance with the Helsinki Agreement and the rules of the Institutional Review Board at the University of Pennsylvania.

### Cerebrospinal Fluid Analyses

CSF samples were obtained by routine lumbar puncture according to standard operating procedures of the Alzheimer's Disease Neuroimaging Initiative (ADNI) (15). In brief, baseline CSF samples were obtained in the morning typically after an overnight fast. Lumbar punctures were performed with a 20- or 24-gauge spinal needle. CSF was collected into polypropylene transfer tubes, 0.5 ml aliquots were prepared from these samples, and then frozen within 1 h. The aliquots were stored in barcode–labeled polypropylene vials at −80°C. Samples were assayed via Luminex for Aß_1−42_, p-tau, and t-tau levels, as previously described (15), and a small number of samples were analyzed by ELISA and transformed to Luminex equivalents using an autopsy-confirmed formula (21).

### Statistical Analysis

In the first analysis, we grouped patients according to the predefined cut-point of 192 pg/ml. Since the cut-point based on an AD phenotype may not generalize to non-amnestic cases with AD pathology, we also implemented a second analytic approach. Here we used a k-means cluster analysis with a two-group solution to identify the groups among all PPA patients in our cohort according to their CSF Aß_1−42_ level, and examined this cut-point in our cohort. The variables utilized in this analysis were the categorical variable “clinical phenotype” and the continuous variable “CSF Aß_1−42_ level.” A receiver operating characteristic (ROC) curve analysis used sensitivity and specificity to define the cut-point between these groups, and provided the area under the curve (AUC). In both the analysis using the predetermined cut-point and the empirically determined cut-point in our cohort, we tabulated the frequency of patients with lvPPA compared to PPA patients with another phenotype in the two CSF-determined groups, and used chi-squared analyses to assess whether there was a statistically significant difference between phenotypes within and between CSF-defined groups.

### MRI Analysis

High resolution T1-weighted MRI scans were available for 20 lvPPA, 10 naPPA, and 11 svPPA, and we compared these PPA patients with 69 demographically matched control participants. The PPA patients with MRI matched the clinical and demographic characteristics of those without MRI (all *p* > 0.1). MRI exclusion criteria included poor-quality MRI at visual inspection (e.g., distortion, excessive motion, or processing failure due to image distortion/artifact). Briefly, participants underwent a structural T1-weighted MPRAGE MRI acquired from a SIEMENS 3.0T Trio scanner with an eight-channel coil using the following parameters: repetition time (TR) = 1,620 ms; echo time (TE) = 3 ms; 160 1.0 mm slices; flip angle = 15°; matrix = 192 × 256; and in-plane resolution = 0.9766 × 0.9766 mm. T1 MRI images were preprocessed using antsCorticalThickness (22). Each individual dataset was deformed into a standard local template space in a canonical stereotactic coordinate system. Registration was performed using a diffeomorphic deformation that is symmetric to minimize bias toward the reference space for computing the mappings and topology-preserving to capture the large deformation necessary to aggregate images in a common space. The ANTs Atropos tool used template-based priors to segment images into 6 tissue classes (cortical gray matter, white matter, CSF, deep gray structures, midbrain, and cerebellum), and generated the probability images of each tissue class (23). Here we focused on cortical gray matter probability (GMP) images that were transformed into MNI space, and downsampled to 2 mm isotropic voxels. This voxel size approximates the true thickness of cortex, although at the cost of less robust *p*-values due to a larger number of comparisons. We smoothed the data using a 2 sigma full-width half-maximum Gaussian kernel before analysis. Voxelwise analyses of GMP were performed using the non-parametric randomize tool implemented in the FMRIB Software Library (FSL: http://fsl.fmrib.ox.ac.uk) with 10,000 permutations that is equivalent to an analysis protecting for multiple comparisons. We report clusters that survived *p* < 0.01 with a minimum of 150 adjacent voxels. We report two sets of *t*-tests: First, we examined each patient group (lvPPA, naPPA, svPPA) relative to controls; and second, we examined each CSF-subgroup relative to controls (low-Aß_1−42_, hi-Aß_1−42_).

## Results

### Identifying Groups Based on CSF Aß_1−42_ Level

A summary of CSF analyte values is provided in Table 1. Twenty-three patients had a low CSF Aß_1−42_ level consistent with likely AD pathology, and 21 (91.3%) of these cases had an lvPPA phenotype, revealing significantly more cases of clinically diagnosed lvPPA than non-lvPPA among PPA patients with a lower CSF Aß_1−42_ level (*p* < 0.001). The sensitivity for low CSF Aß_1−42_ level to identify lvPPA compared to non-lvPPA is 91%; the specificity is 89%; the positive predictive value is 87%; and the negative predictive value is 92%. Of the 2 non-lvPPA cases with a lower Aß_1−42_ level, one had an svPPA phenotype (CSF Aß_1−42_ level = 167 pg/ml) and the other had a naPPA phenotype (CSF Aß_1−42_ level = 183 pg/ml). The svPPA patient with CSF Aß_1−42_ level < 192 pg/mL had an age at onset of 68. Not only was this well-above the mean age of onset of lvPPA patients with CSF Aß_1−42_ < 192 pg/mL, but it was also well above the mean age at onset of the svPPA patients with CSF Aß_1−42_ level > 192 pg/mL (*M* = 60.18 years, SD = 7.45), suggesting the possibility of AD co-pathology. Duration of disease at the time of obtaining CSF was shorter in this svPPA patient (1 year) and longer in this naPPA patient (7 years), and differed from the mean disease duration of lvPPA patients with CSF Aß_1−42_ < 192 pg/mL (*M* = 2.7 years, SD = 1.6) and from that of PPA patients with CSF Aß_1−42_ level >192 pg/mL (*M* = 2.57 years, SD = 1.65). The svPPA and naPPA patients with CSF Aß_1−42_ < 192 pg/mL levels did not differ from lvPPA patients with CSF Aß_1−42_ < 192 pg/mL, and did not differ from PPA patients with CSF Aß_1−42_ level >192 pg/mL with regards to education level.

Twenty-one (80.8%) of 26 cases with a clinical diagnosis of lvPPA had a CSF Aß_1−42_ level < 192 pg/mL, significantly greater than the number of lvPPA cases with CSF >192 pg/mL (*p* < 0.001). The sensitivity for lvPPA to identify low CSF Aß_1−42_ level compared to elevated CSF Aß_1−42_ level is 81%; the specificity is 75%; the positive predictive value is 91%; and the negative predictive value is 78%. The mean CSF Aß_1−42_ level of the 5 lvPPA cases with CSF >192 pg/mL was 317 pg/mL (SD = 186). The lvPPA patients with CSF Aß_1−42_ < 192 pg/mL did not differ significantly from those with CSF Aß_1−42_ level >192 pg/mL with regards to education level and disease duration at the time that CSF was obtained. The age at onset, however, differed significantly [*t*_(22)_ = 4.20, *p* < 0.001], with the 5 lvPPA patients with an elevated CSF Aß_1−42_ level having an older age at onset (*M* = 65.2 years, SD = 9.36) than lvPPA cases with CSF Aß_1−42_ < 192 pg/ml (*M* = 58.87, SD = 7.23).

Since CSF Aß_1−42_ level in non-amnestic AD with early-onset disease may differ from the CSF Aß_1−42_ level associated with later-onset amnestic AD, we also used a cluster analysis to partition the entire cohort of PPA patients (*n* = 46) according to the CSF Aß_1−42_ level. One cluster included PPA patients with a lower Aß_1−42_ level (*n* = 23, *M* = 145.2 pg/ml; SD = 27.8), and the second cluster included PPA patients with a higher Aß_1−42_ level (*n* = 23, *M* = 344.1 pg/ml; SD = 159.4). A ROC curve analysis in this sample defined a cutpoint at 204.2 pg/mL, yielding 91% sensitivity, 89% specificity, and an area under the curve = 0.914. Independent samples *t*-test showed that the Aß_1−42_ level of the cohort with likely AD pathology to be significantly lower than that of the cohort less likely to have AD pathology [*t*_(44)_ = 6.6, *p* < 0.001].

### MRI Imaging

**Figure 1:**
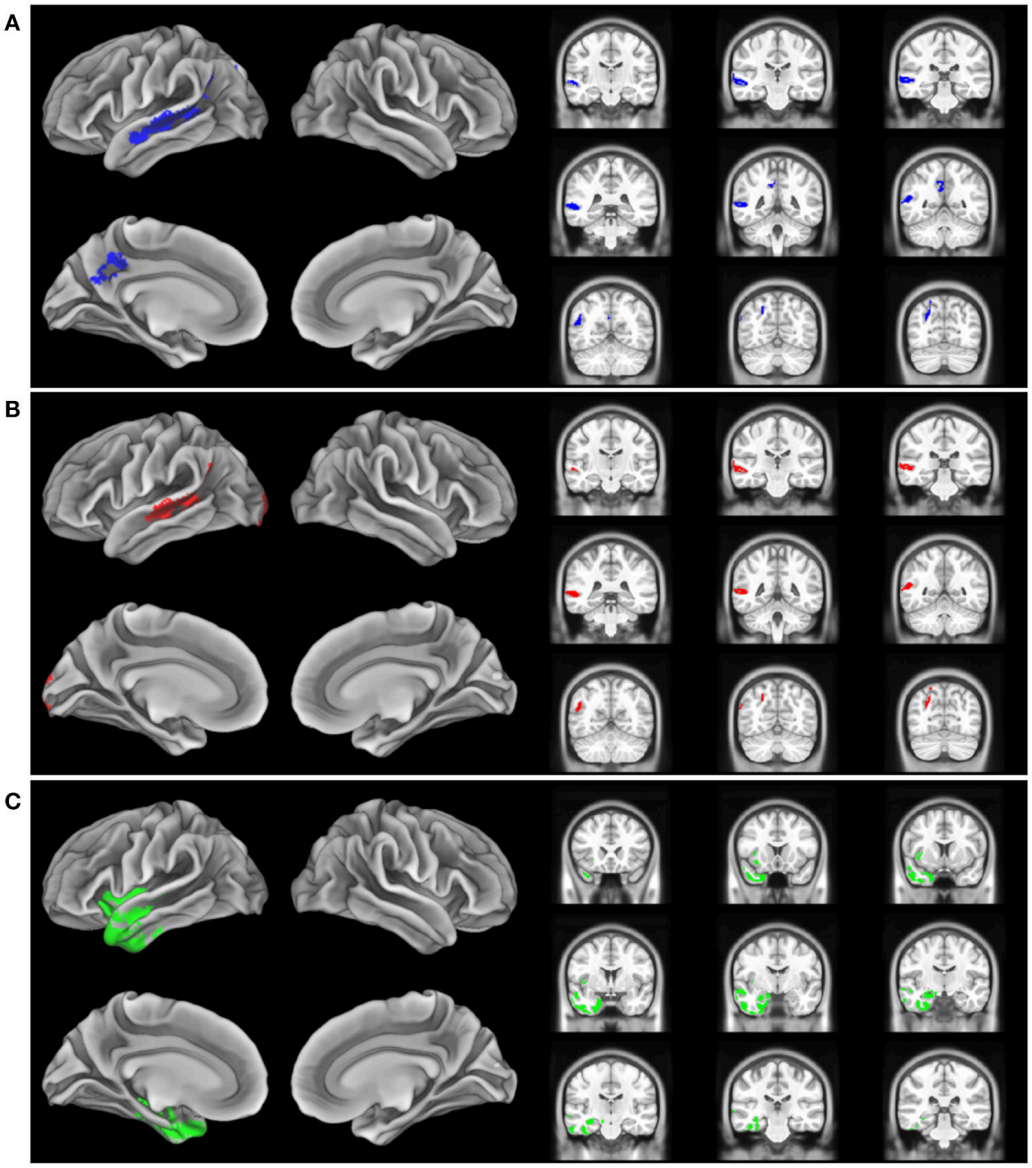
Distribution of significant gray matter atrophy in T1-weighted MRI scans. **(A)** low CSF Aß1-42 (<192 pg/mL) vs. controls, **(B)** logopenic variant PPA vs. controls, **(C)** High CSF Aß1-42 (>192 pg/mL) vs. controls.

The imaging analysis evaluated the anatomic distribution of disease in the cohort of subjects with a low Aß_1−42_ level and a high Aß_1−42_ level. This demonstrated distinct areas of atrophy that stratified the two groups (Figure 1; Table 3). Significant areas of GM atrophy for the low Aß_1−42_ cohort were in the left middle-superior temporal gyrus, left parietal region, and left precuneus (Figure 1A). This overlapped substantially with the analysis of the cohort with a lvPPA phenotype where significant atrophy was found in the left middle-superior temporal gyrus, left parietal region, and left occipital region (Figure 1B). Neither group had significant hippocampal atrophy, emphasizing the PPA phenotype as opposed to a language-dominant syndrome of clinical AD. By comparison, in the high Aß_1−42_ cohort, significant atrophy was found in the left anterior temporal region and left inferior frontal-insula region *p* < 0.01, k = 150 (Figure 1C), anatomic areas associated with svPPA and naPPA, respectively.

**Table 3:** MRI atrophy in patient groups relative to healthy controls, and in comparisons of patient groups, including cluster size, coordinates, anatomic location, and Brodmann area.

**Cluster size**	**Peak x-coord**	**Peak y-coord**	**Peak z-coord**	**Anatomical location**	**BA**
**Low Abeta** **<** **Controls**					
912	−50	−4	−16	Left superior temporal gyrus	22
206	−28	−84	20	Left superior occiptal gyrus	19
150	−4	−56	28	Left posterior cingulate	31
**LvPPA subgroup-Low Abeta** **<** **Controls**					
772	−50	−24	−8	Left middle temporal gyrus	21
189	−20	−98	−16	Left lateral occiptal gyrus	18
183	−32	−90	20	Left superior occiptal gyrus	19
**High Abeta** **<** **Controls**					
2,067	−38	−6	−48	Left inferior temporal gyrus	20
158	−30	20	−12	Left inferior orbitofrontal gyrus	47

*lvPPA, logopenic variant primary progressive aphasia; naPPA, non-fluent/ agrammatic variant primary progressive aphasia; svPPA, semantic variant primary progressive aphasia; low Aß_42_ = Aß_42_ level < 192 pg/mL); high Aß_42_ = Aß_42_ level >192 pg/mL). All analyses p < 0.01, k = 150; please see text for details*.

## Discussion

Autopsy studies have demonstrated that the three PPA variants—lvPPA, svPPA, and naPPA—are often associated with distinct underlying pathology (2, 3). In an era of expensive diagnostic markers such as molecular PET and the advent of disease-modifying treatment trials targeting a specific pathology, it is valuable to have less expensive biomarkers that can screen for underlying pathology. However, identifying patients with likely AD pathology during life has been particularly challenging: While the lvPPA phenotype was developed in part to identify the subgroup of PPA patients with likely AD pathology, the clinical criteria for lvPPA have proven to be relatively less reliable (4–7, 12). Here we examined the usefulness of a reliable and valid proxy of AD pathology—CSF analytes—to identify a subset of PPA cases with a phenotype that may be associated with AD pathology. Our findings suggest that many PPA patients with a low CSF Aβ_1−42_ level have a lvPPA phenotype.

We found that a low Aβ_1−42_ level (< 192 pg/mL) is present in the CSF of many PPA patients, suggesting that these PPA patients may have AD pathology (15). It is potentially valuable that most of these patients had a phenotype most consistent with lvPPA. The criteria defining lvPPA have been controversial (4–7). In our study, we accepted a lvPPA phenotype as defined by a clinical-pathological study (14), which included word-finding difficulty together with a deficit of repetition marked by a low forward digit span score. We found that a very large proportion of cases with a CSF Aß_1−42_ level in the range consistent with likely AD pathology have a clinical phenotype of lvPPA.

While a large majority of cases with a low CSF Aß_1−42_ level had lvPPA, this phenotype alone cannot be used reliably to identify patients with likely AD pathology. For example, in our cohort we found two patients with a low CSF Aß_1−42_ level who did not have a lvPPA phenotype: One patient had svPPA and another had naPPA. Without autopsy evidence, we can only speculate about the basis for this discrepancy. One possibility is that these cases in fact have AD pathology but in an anatomic distribution more consistent with these non-lvPPA phenotypes. Several patients with atypical presentations of AD pathology have been reported with svPPA or naPPA phenotypes (24, 25). A second possibility is that these cases may have secondary AD co-pathology in the context of primary FTLD pathologies causing these syndromes. Co-pathology is not uncommon in neurodegenerative disease (26), and we found in our autopsy series that CSF analytes for AD are significantly biased by the presence of AD co-pathology even in individuals where the primary pathology is consistent with an FTLD spectrum pathology (23).

Another important consideration is that 26 cases in our cohort had a lvPPA phenotype, but only 21 of these cases with a lvPPA phenotype had low CSF Aß_1−42_. The five lvPPA cases with elevated CSF Aß_1−42_ levels had an age of onset that was older than that of the lvPPA patients with lower CSF Aß_1−42_ levels. With the caveat that we did not have a pathologic diagnosis in our cases, our findings are consistent with the claim that a clinical evaluation for lvPPA can be an inexpensive way to screen PPA patients for those who may be eligible for participation in disease-modifying therapies targeting the misfolded proteins contributing to AD pathology. Additional biomarker data would be helpful to confirm the diagnosis of PPA associated with likely AD such as amyloid-PET, although it should be noted that amyloid-PET is also associated with false-positive and false-negative findings (27).

The MRI analysis was consistent with the finding that the lvPPA phenotype is most prominent in the low Aβ_1−42_ level cohort by showing nearly identical areas of reduced GMP in the lvPPA cohort and the low Aβ_1−42_ level cohort (Figures 1A,B). The pattern of atrophy of the cohort with the lvPPA phenotype included in the left middle-superior temporal gyrus, left parietal region, and left occipital region. This resembles the distribution of MRI atrophy seen in other MRI studies of lvPPA (13, 14, 26, 28). Atrophy in the left lateral temporal lobe is associated with lexical retrieval (29) and auditory-verbal short-term memory (13) that are compromised in lvPPA. Moreover, this closely resembles the areas of GM atrophy in the low Aß_1−42_ cohort, including the left middle-superior temporal gyrus, left parietal region, and left precuneus. This anatomic distribution of atrophy is a subset of regions typically affected in clinical AD. Importantly, our cohort did not have significant medial temporal lobe atrophy, emphasizing that these patients had PPA and not a language-dominant variant of AD. Despite the small cohort of PPA patients with elevated CSF Aβ_1−42_ consistent with a non-AD form of PPA, the pattern of atrophy in this group is distinctly different from that of the low CSF Aβ_1−42_ cohort with lvPPA. The areas of atrophy found in the group with elevated CSF Aβ_1−42_ include the left anterior temporal region, and the left inferior frontal-insula region. These are areas associated with svPPA and naPPA, respectively (28, 30).

Other groups have explored the utility of using CSF analytes to differentiate PPA phenotypes. One study evaluated CSF levels of Aß_1−42_, t-tau, and p-tau_181_ in a small cohort of PPA patients, AD patients, and healthy controls (16). They found that the ratio of p-tau_181_/Aß_1−42_ ratio allowed separating AD and non-AD patients, although there was no available converging evidence such as imaging or autopsy to support this finding. Another study also found lower levels of CSF Aβ_1−42_ in clinically-diagnosed lvPPA compared to other PPA patients, discriminating between lvPPA and naPPA/svPPA with 86% sensitivity and 69% specificity (22). In a small autopsy series from Northwestern University, six of nine autopsied PPA cases with AD pathology had an antemortem proprietary CSF ATI score in the range consistent with Alzheimer's disease pathology (23). In a large, multi-center cohort of PPA patients with pathology determined by CSF or PET molecular markers or autopsy findings, 86% of lvPPA patients had biomarker findings consistent with Aß pathology (17). An important challenge to the use of CSF or PET biomarkers in the present study and this previously published work is that co-pathology is frequently present in neurodegenerative disease (26, 31). In particular, AD co-pathology may be present in cases with other primary pathologies, and thus give the false impression that a patient's primary pathologic diagnosis is AD. In the study of Bergeron et al. (17), for example, primary AD pathology was present only in 76% of cases, and the discrepancy between pathology ascertained at autopsy compared to pathology estimated by biomarkers may have been due in part to the sensitivity to secondary AD co-pathology in CSF biomarker-ascertained cases with non-AD primary pathologies. Neurogranin (Ng) also has been identified as a CSF biomarker associated with AD pathology, and we found that Ng is significantly elevated in a clinical cohort of lvPPA patients that partially overlaps with the cohort presented in the current study (24). Serum neurofilament light chain (NfL) also may be useful for discriminating between lvPPA and non-lvPPA with 81% sensitivity and 67% specificity (22), and others have shown elevated NfL in svPPA and naPPA relative to a small number of lvPPA (25) [also see (31)], although others have found CSF NfL elevated in AD (32, 33). Considerable caution must be adopted in concluding from screening studies that lvPPA may be a marker for AD pathology: Our study and others suggest that lvPPA may be associated with a CSF surrogate for AD pathology, but this does not exclude the possibility that other pathologies may be contributing to a patient's difficulties.

Several caveats should be kept in mind when considering our results. First, a relatively small number of patients participated in our study, although PPA is a relatively rare condition, and we adhered narrowly to the published criteria for PPA to determine the usefulness of AD CSF analytes within the scope of these criteria. Other CSF analytes may be informative in PPA and have been reported in some patients from this cohort elsewhere (24), and additional work is needed to assess these other analytes. Very few autopsy-validated studies of CSF analytes have been reported including some of the patients from this study (17, 23), and although we used autopsy-validated CSF analytes, another limitation is that we knew the true pathologic diagnosis in only a very small number of these PPA cases. Additional work is needed with an autopsy-defined cohort (14). lvPPA has been associated with cerebral microbleeds {Mendes:vv}, although there were no cerebral microbleeds in our cohort. Generalizeability of our findings may be limited since this is a single-center study. With these caveats in mind, this study demonstrated that low CSF Aβ_1−42_ is not uncommon in patients with PPA, and that there is a statistical association between a low CSF Aβ_1−42_ level and the lvPPA phenotype. The link between lvPPA phenotype and a surrogate marker of AD pathology was further supported by MRI imaging. The potential use of the lvPPA clinical phenotype to screen for CSF analytes as a surrogate for likely AD pathology may help establish eligibility of these patients for disease-modifying treatment trials.

## Ethics Statement

This study was carried out in accordance with the recommendations of the Institutional Review Board of the University of Pennsylvania with written informed consent from all subjects and witnessed by a responsible caregiver. All subjects gave written informed consent in accordance with the Declaration of Helsinki. The protocol was approved by the Institutional Review Board of the University of Pennsylvania.

## Author Contributions

AH, CJ, MU, DI, and MG data collection. CN, CJ, CM, and KC data analysis. CN, CM, DI, KC, and MG manuscript writing and editing.

### Conflict of Interest Statement

MG is an Associate Editor of Neurology, participates in clinical trials sponsored by Biogen, Alector, and Eisai, and receives support for his role as a consultant for Biogen, UCB, Ionis and Bracco. The remaining authors declare that the research was conducted in the absence of any commercial or financial relationships that could be construed as a potential conflict of interest.
